# Electrochemically modulated single-molecule localization microscopy for *in vitro* imaging cytoskeletal protein structures

**DOI:** 10.1515/nanoph-2024-0559

**Published:** 2025-02-10

**Authors:** Chenghong Lei, Dehong Hu

**Affiliations:** College of Chemistry and Bioengineering, 66515 Guilin University of Technology, Guilin, Guangxi 541006, China; Environmental Molecular Sciences Laboratory, Pacific Northwest National Laboratory, Richland, WA 99352, USA

**Keywords:** single-molecule localization imaging, electrochemical modulation, redox-active fluorophores

## Abstract

A new concept of electrochemically modulated single-molecule localization super-resolution imaging is developed. Applications of single-molecule localization super-resolution microscopy have been limited due to insufficient availability of qualified fluorophores with favorable low duty cycles. The key for the new concept is that the “On” state of a redox-active fluorophore with unfavorable high duty cycle could be driven to “Off” state by electrochemical potential modulation and thus become available for single-molecule localization imaging. The new concept was carried out using redox-active cresyl violet with unfavorable high duty cycle as a model fluorophore by synchronizing electrochemical potential scanning with a single-molecule localization microscope. The two cytoskeletal protein structures, the microtubules from porcine brain and the actins from rabbit muscle, were selected as the model target structures for the conceptual imaging *in vitro*. The super-resolution images of microtubules and actins were obtained from precise single-molecule localizations determined by modulating the On/Off states of single fluorophore molecules on the cytoskeletal proteins via electrochemical potential scanning. Importantly, this method could allow more fluorophores even with unfavorable photophysical properties to become available for a wider and more extensive application of single-molecule localization microscopy.

## 1 Introduction

In the past more than 20 years, super-resolution optical microscopies have been rapidly developed, where single-molecule localization microscopy (SMLM) is remarkable as it allows the optical resolution to reach a few to tens of nanometers in studying complex biological systems [1], [2], [3], [4], [5], [6], [7], [8]. The SMLM techniques have been extensively used for super-resolution imaging in living cells and tissues [9], [10], [11], [12], [13]. There are a few developed techniques for SMLM including PhotoActivated Localization Microscopy (PALM), Fluorescence PhotoActivated Localization Microscopy (FPALM) [1], [2], [3], [4], and Stochastic Optical Reconstruction Microscopy (STORM) [5], [6], [7], [8], where PALM and FPALM normally used fluorescent proteins as fluorophores, STORM employed synthetic fluorescent dyes. The key to obtain SMLM images is based on the temporal separation of actively fluorescing molecules and the position of these sparse molecules can be located much more precisely than their diffraction limits over ∼200 nm. Among SMLM techniques, a set of laser beams are used to stochastically activate and modulate the emission of the sparse fluorescent molecules on a specific biological system, making those separate (single) fluorescent molecules blinking from time to time and thus be precisely localized. The sum of thousands of recordings of the precise localizations of single fluorescent molecules over a time window turns out the highly resolved images of the target biological structures [14].

SMLM requires a large number of spatially resolved single-molecule images with high signal-to-noise contrast. There are many requirements for preferred fluorophores for SMLM [9], [15]: The fluorophores labeled on the samples must have the capability of “On” and “Off” blinking, that is, photoswitching between bright and dark fluorescent states; the contrast between the intensities of the bright (On) and dark (Off) states of the fluorophores must be very high; there would also be strict criteria for special photophysical properties of the fluorophores, such as duty cycle (defined as fraction of time of the fluorophore spent at “On” state prior to photobleaching), number of emitted photons per localization event, blinking times, photobleaching time, and others [16]. To get enough single-molecule localizations to represent the target structure with super resolution, the coverage of fluorophore molecules on the target structure cannot be too low. On the other hand, to avoid data error in localizing single molecules, when one molecule is at “On” state, all other molecules nearby need to be at “Off” state. If two molecules are at “On” state within the proximity of diffraction limit, the middle point of the two molecules would be mistakenly called as the location of a “merged” molecule. This causes error in localizing the two molecules. To avoid this error, there are basic approaches: (1) reduce labeling density of the dye; (2) choose low duty cycles of dye molecules. Reducing dye molecule density in many cases is not a good solution because sparse dye molecules cannot present the fine and complex biological structure to be imaged [15], which make the imaging much less meaningful. Therefore, people would like to choose low duty cycles of dye molecules for super-resolution SMLM imaging. Several papers have listed those preferred fluorophores used for SMLM imaging with duty cycles in the range of 1.0 × 10^−4^–1.0 × 10^−3^ for practically minimizing these localization errors [9], [15], [16], [17]. In fact, although most of single molecules of fluorophores exhibit the phenomena of fluorescence blinking [18], many of them have too high duty cycles, not suitable for SMLM imaging. The localization-based image from high duty cycle molecules could be distorted because of the localization errors from molecule overlapping [16]. The duty cycle is an intrinsic photophysical property of the fluorophore, and it is difficult to improve. With these criteria in mind, the number of qualified photoswitchable fluorophores for SMLM imaging is very limited.

**Scheme 1:**
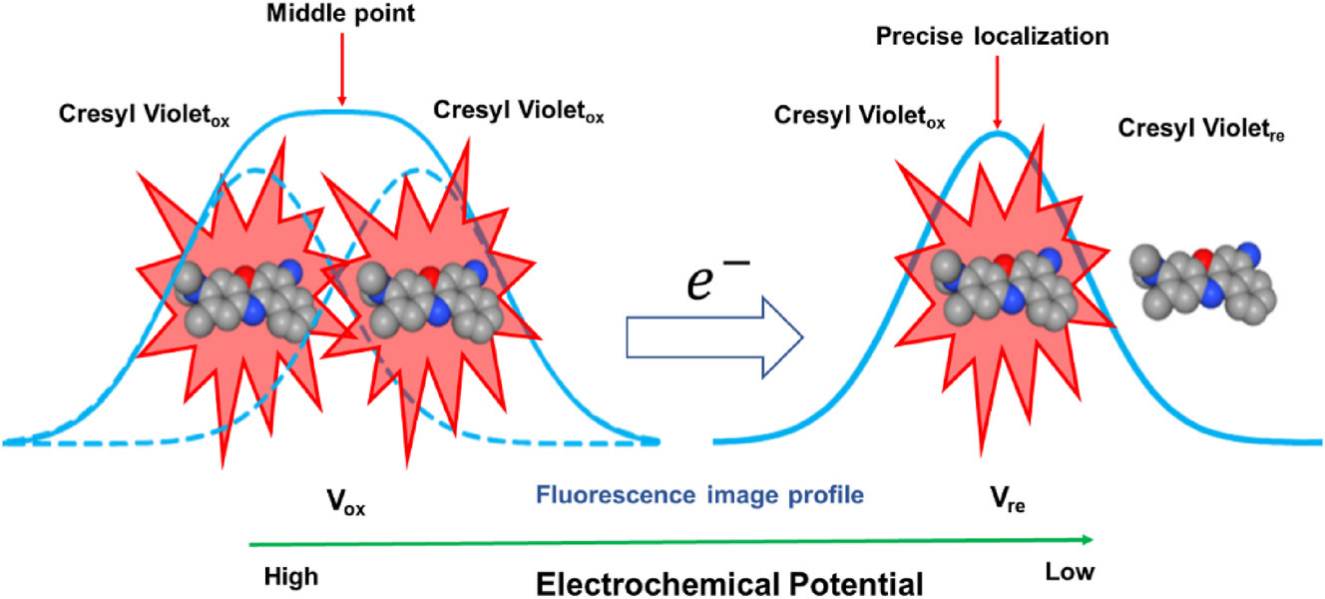
Avoiding localization error by electrochemical modulation: one of two oxidized cresyl violet molecules within diffraction limit is electrochemically driven from “On” (oxidized, ox) state to “Off” (reduced, re) state while the electrochemical potential is scanning from high (*V*_ox_) to low (*V*_re_).

In this work, we are developing a new concept of electrochemically modulated single-molecule localization super-resolution imaging by electrochemical switching of “On/Off” states of some redox-active fluorophores for SMLM. So far, there has been rarely a report that a redox-active fluorophore with unfavorable blinking kinetics was ever used in SMLM imaging with the help of electrochemical control. The key for the new concept is that the “On” state of a redox-active fluorophore with unfavorable high duty cycle could be driven to “Off” state by electrochemical potential modulation and thus become available for SMLM imaging. We demonstrated the new concept with electrochemically modulated STORM (stochastic optical reconstruction microscopy) (E-STORM) by *in vitro* imaging of two cytoskeletal protein structures microtubules and actins using a redox fluorescent dye cresyl violet, whose duty cycle was estimated to be 0.18. Although the duty cycle of cresyl violet is its intrinsic value that is hard to change or improve, the major reason for cresyl violet to be selected as a model redox fluorophore for this work lies in that cresyl violet can be electrochemically driven from an oxidized fluorescent “On” state to a reduced nonfluorescent “Off” state [19], [20]. The electrode reaction is proposed as [19]:

(1) $$\begin{array}{ll} & \text{Cresyl}\;\text{violet}\left(\text{fluorescent}\right)\\ & \;\overset{+\text{e}}{\underset{-\text{e}}{⇌}}\;\text{Cresyl}\;\text{violet}\left(\text{non }-\text{ fluorescent}\right) \end{array}$$

which could allow cresyl violet to be an excellent candidate of redox fluorophore for electrochemically overcoming the disadvantage of high duty cycle as well as for exhibiting the best contrast of bright/dark states for single-molecule localization. Although cresyl violet at oxidized state has the duty cycle of 0.18, much larger than other STORM dyes such as Alexa 647 [9], [15], [16], [17], cresyl violet at reduced state has no fluorescence. The experimentally observed percentage of time that a cresyl violet molecule emitting fluorescence is related to its inherent duty cycle as well as the percentage of time at oxidized state. Under electrochemical potential scanning and the excitation laser beam with a constant intensity, the fluorescing molecules of cresyl violet on microtubules and actins displayed temporally modulated emission of the sparse fluorophores periodically and synchronously in the same cycle as the electrochemical potential scanning, where the spontaneous blinking of single fluorescent molecules still accounted for a small fraction of emission changes [21], [22], [23], [24]. Since the “On/Off” state of the redox fluorescent cresyl violet molecules were able to be electrochemically modulated, not only the fluorophore’s blinking kinetics were much improved but also the dark states of the fluorophore were well controlled allowing the molecule overlapping and the thus localization errors to be avoided or reduced at most. Scheme 1 shows that one of the two fluorescing molecules of cresyl violet within the proximity of diffraction limit is able to be precisely located since the other molecule can be electrochemically reduced to be nonfluorescent. Accordingly, the localization error, which says the middle point of the two molecules as the location of a “merged” molecule (Scheme 1), would be avoided. The high-quality images of microtubules and actins were thus able to be produced from the rendering of the precisely determined localizations of single molecules of cresyl violet within the time (potential) window when electrochemical potential modulation reduced the crowding of single molecules, even though cresyl violet’s duty cycle is high. Our results also demonstrated that the electrochemical potential modulation could provide an indicator whether overcrowding of single molecules occurred. In contrast, without electrochemical modulation, distorted images of the target protein structure were obtained due to localization errors. Several more classes of redox fluorophores including dyes of phenazines, phenoxazines, and phenothiazines may be utilized in E-STORM. This work may open up a new approach for STORM and other SMLM techniques for a variety of applications.

## 2 Experimental section

Microtubules (MT002-A) from porcine brain, muscle actin from muscle (AKL99), and paclitaxel (Taxol, TXD01) were obtained from Cytoskeleton Inc. The typical length of microtubule is ∼2 µm as the product manual specified. The microtubule stock solution was diluted to 0.2 mg/mL in the working buffer, pH 7.0, 15 mM PIPES containing 1 mM MgCl_2_. Taxol of 20 µM was used for preserving the structure of microtubules. The muscle actin has an approximate molecular weight of 43 kDa. About 1.0 mg/mL of the actin stock solution was prepared in pH 8.0, 5 mM Tris-HCl containing 0.2 mM CaCl_2_. The polymerization buffer for actin contained 0.5 M KCl, 20 mM MgCl_2_, and 10 mM ATP. A concentration of 0.36 mg/mL of the polymerized actin was prepared in the diluted polymerization buffer of pH 8.0, 4.5 mM Tris-HCl containing 0.18 mM CaCl_2_, 0.91 mM ATP, 1.81 mM MgCl_2_, and 45.45 mM KCl. And 36 μM of cresyl violet (SigmaAldrich) in H_2_O was used for preparing various diluted cresyl violet solutions in the working buffer. About 0.0–9.0 nM cresyl violet was used to stain microtubules in the working buffer. Indium tin oxide (ITO)-coated glass coverslip (22 × 22 mm, 8–12 Ω resistance) was used as the base electrode throughout this work. For the measurement of the duty cycle of cresyl violet, the Nafion-modified ITO surface, where single molecules of cresyl violet were adsorbed, was prepared as previously reported [25]. About 200 µL of the working solution was used inside 10 cm of vinyl tubing (3/8 inch OD and 1/4 inch ID) as the electrochemical cell attached to the ITO surface by epoxy glue.

All electrochemical experiments and the experiments coupled with single-molecule localization super-resolution fluorescence microscope were carried out with a potentiostat (CH instrument, USA). The experimental setup for the super-resolution fluorescence microscope was described previously [26]. The experiments were performed on an inverted fluorescence microscope (Olympus IX71), equipped with a 100X NA 1.4 oil immersion objective (Olympus UPlanSApo) [26]. The potentiostat was connected to the electrochemical cell using ITO as the working electrode, a Ag/AgCl (1.0 M KCl) electrode (CH Instrument, CHI111P) as the reference electrode and a platinum wire coil as the counter electrode [25]. The electrochemical cell held 200 μL of the working solution above an exposed ITO surface area of 31.65 mm^2^ [25]. All experiments and experimental preparations were carried out at ambient conditions at room temperatures (22 ± 1 °C). A 594 nm wavelength laser (Coherent OBIS) was brought to the super-resolution fluorescence microscope’s epi-port by mirrors. The excitation power used for imaging was 6 mW. The excitation was reflected by a dichroic mirror (Di02-R594-25 × 36, Semrock). The emission passed through this dichroic mirror and emission band pass filter (FF01-647/57-25, Semrock). The fluorescence images of single molecules on the ITO surface were recorded using an EMCCD camera (Andor iXon ultra 897). The images were acquired continuously at exposure rate of 0.2 s per frame throughout CV potential scanning and the control experiments. The camera sent a trigger signal to the potentiostat for simultaneous recording of both imaging signal and electrochemical signal. Our Matlab program processes every image with registered electrochemical potential to track every molecule’s location. The location of single molecules is calculated based on Gaussian mask method [27]. This is a well-established method of calculating the position of a single molecule based on the center-of-mass of its image spot. Our Matlab program based on this method has been used in our previous publications [26], [28].

## 3 Results and discussion

**Figure 1:**
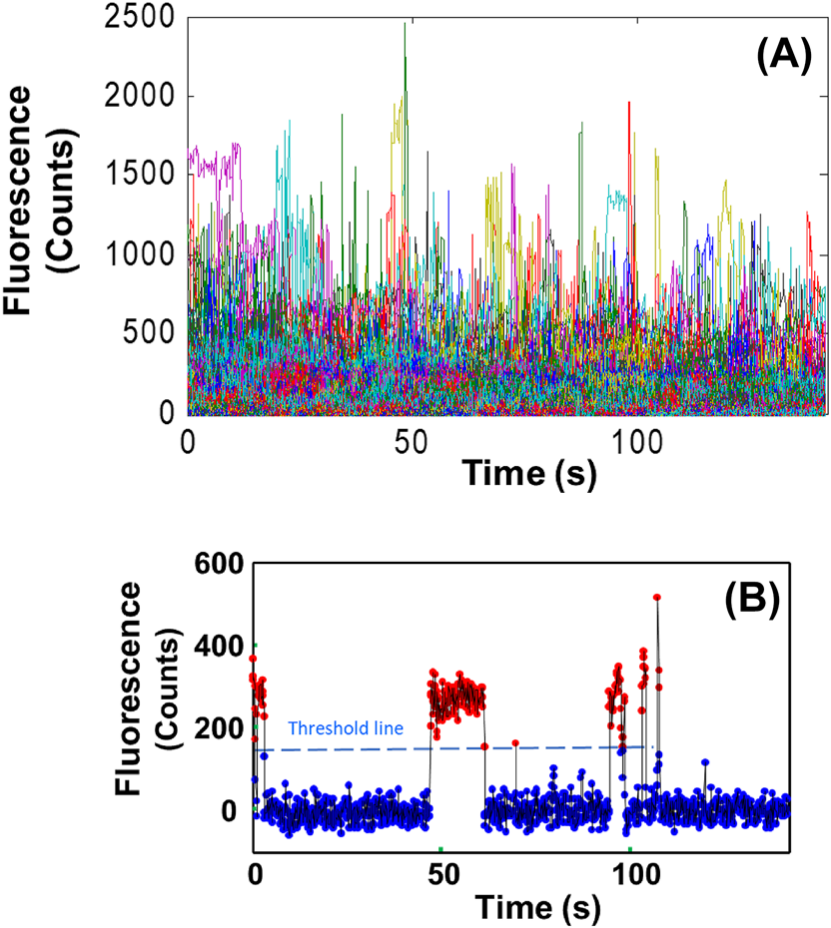
Fluorescence intensity-time trajectories for calculation of the duty cycle of cresyl violet. (A) Fluorescence intensity-time trajectories of 717 single molecules of cresyl violet on the Nafion-modified ITO surface without electrochemical modulation; (B) the fluorescence-time trajectory of one representative single molecule of cresyl violet from (A) for calculation of the duty cycle of cresyl violet, where the dashed line is the On/Off threshold line for the “On” time (red dots) and the “Off” time (blue dots) of the molecule. The working buffer: pH 6.2, 5 mM sodium phosphate.

Cresyl violet is a strongly fluorescent and redox-active cation dye [29], which has been used for conventional staining [30], [31], [32], [33], and single-molecule spectroelectrochemistry [19], [20], [25]. The duty cycle, as the fraction of time of a single fluorescent molecule at “On” state, is an important parameter to describe single-molecule blinking behavior. Molecules with too high duty cycles are not suitable for using for SMLM imaging because it is difficult to obtain well separated single molecules in an image. We have examined the single molecule intensity trajectories of 717 single molecules of cresyl violet adsorbed on the Nafion-modified ITO surface without electrochemical modulation and thereby cresyl violet’s duty cycle was estimated to be 0.18 (Figure 1A and B). The apparent duty cycle of cresyl violet with the electrochemical potential scanning, based on the previous data of 612 molecules on the same electrode surface [25], was ∼0.12. These numbers are orders of magnitude larger than many commonly used dyes for SMLM imaging, which are typically in the range of 1.0 × 10^−4^–1.0 × 10^−3^ [9], [15], [16], [17]. Therefore, without electrochemical modulation, cresyl violet is unsuitable to be used for SMLM imaging.

Cyclic voltammetry (CV) is a powerful electrochemical method to investigate the reduction and oxidation processes of molecular species [34], [35]. A potentiostat, providing CV potential scanning, was synchronously triggered by the high-sensitivity and high-speed camera of single-molecule localization microscope (*Experimental Section*). The single-molecule localization super-resolution fluorescence microscope was used in this work for its wide-field imaging capability for simultaneously recording fluorescence intensities trajectories of hundreds of single molecules on the imaging area under electrochemical potential modulation. The fluorescence intensity of single molecules can spontaneously fluctuate between high and low levels, which is often called single molecule blinking “On” and “Off.” These blinking events might be the result of photo-induced charge transfer [21], intersystem crossing to triplet state [22], and chemical reactions [23], [24], which is a ubiquitous phenomenon in single molecule fluorescence even without electrochemical potential modulation. However, cresyl violet is redox active. Under electrochemical (CV) potential scanning, cresyl violet’s fluorescent state transits from bright to dark when electrochemical reduction occurs, and from dark to bright when electrochemical oxidation occurs [19]. In this situation, although the spontaneous blinking still occurs, the blinking On/Off events were mainly the consequence of CV-modulated redox reaction of single molecules of cresyl violet.

**Figure 2:**
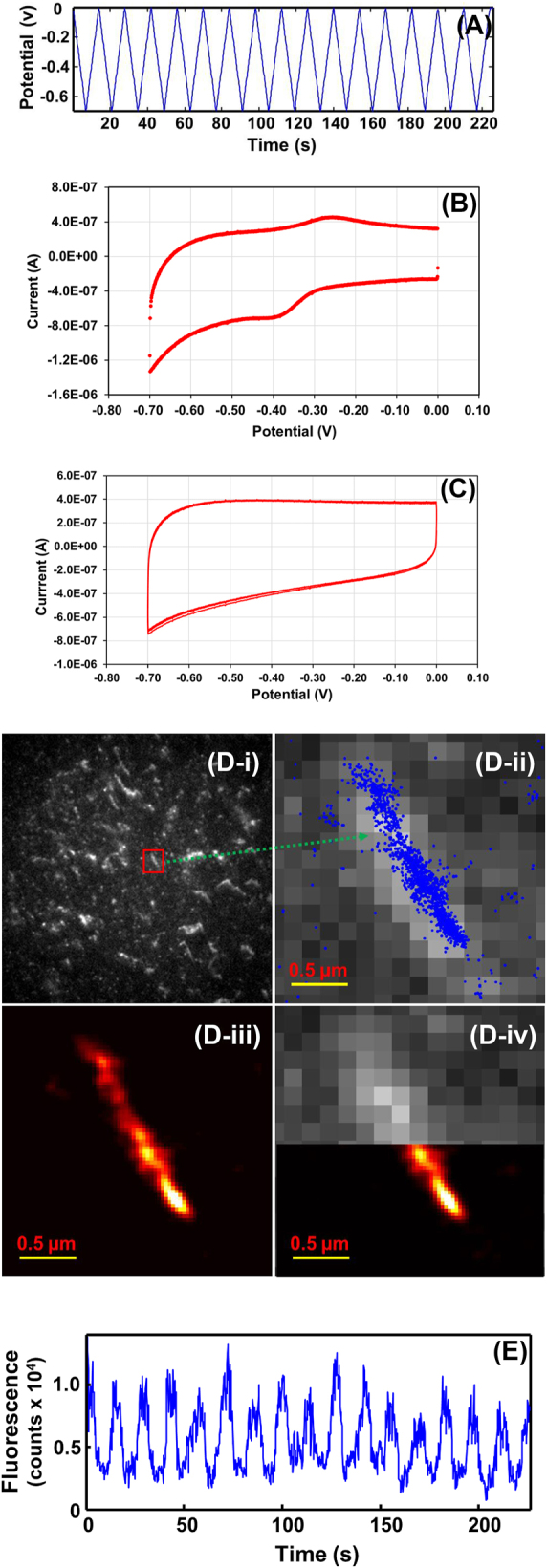
Fluorescence images and intensity-time trajectories of microtubule-cresyl violet with cyclic electrochemical potential scanning at the ITO electrode in the working buffer. (A) Plot of potential (*V*) versus time of 16 continuous CV scans; (B) cyclic voltammograms of 5.1 μM cresyl violet at the ITO electrode in the working buffer; (C) cyclic voltammograms of 3.6 nM cresyl violet and 0.2 mg/mL microtubules at the ITO electrode in the working buffer; (D) the wide-field fluorescence image of microtubule-cresyl violet of a focused area on the ITO electrode with electrochemical modulation (Supplementary Video, Movie S1, Supplementary Information) (i); one representative microtubule selected, where precise localizations of cresyl violet stained on microtubules are plotted as blue dots (ii) (Supplementary Video, Movie S2, Supplementary Information), the resulting super-resolution image of the microtubule from rendered single-molecule positions (iii), and comparison of one half imaged in conventional microscopy and another half imaged in E-STORM to show the resolution improvement (iv); (E) plot of fluorescence intensity of microtubules-cresyl violet displayed in (D-ii) versus time during 16 continuous CV scans. The working buffer: 200 μL, pH 7.0, 15 mM PIPES containing 1 mM MgCl_2_ and 20 µM Taxol. [microtubule]: 0.2 mg/mL; [cresyl violet]: 3.6 nM; scan rate: 0.1 V/s; scan numbers: 16 cycles; potential scan range: 0.0 V to −0.7 V.

Microtubules in the working buffer in the presence of Taxol and actins in the diluted polymerization buffer were incubated with cresyl violet in the lab-made electrochemical cell (*Experimental Section*), where microtubules and actins were spontaneously stained by cresyl violet. The fluorescence of microtubules and actins stained with cresyl violet on the ITO electrode surface was imaged by laser excitation at 594 nm wavelength. Figure 2A shows 16 cycles of cyclic voltammetric potential scanning was applied using ITO glass cover slip as the working electrode in the working solution at a scan rate of 0.1 V/s over the scanning range from 0.0 V to −0.7 V (vs. Ag/AgCl) (*Experimental Section*). When the concentration of cresyl violet was 5.1 μM in the working buffer of pH 7.0, 15 mM PIPES containing 1 mM MgCl_2_ and 20 µM Taxol, cresyl violet can display well-defined cyclic voltammograms at the ITO base electrode with the reduction peak potential at −390 mV and the oxidation peak potential at −272 mV, resulting in a formal potential of −331 mV and a Δ*Ep* value (the difference between reduction and oxidation peaks) of 118 mV under CV potential scanning at a scan rate of 100 mV/s (Figure 2B). The results imply that cresyl violet displayed a quasi-reversible cyclic voltammetric behavior and the electron transfer between cresyl violet and the ITO electrode surface was fast. As expected, when the concentration of cresyl violet was diluted down to as low as 3.6 nM in the working solution containing 0.2 mg/mL microtubules, the cresyl violet molecules were electrochemically undetectable due to the relatively low concentration of the dye as expected (Figure 2C). Nonetheless, the fluorescence intensities of cresyl violet on the imaging area were modulated synchronously with CV potential scanning as discussed later in this work (Movie S1, Supplementary Information), indicating the redox reactions of cresyl violet on electrode occurred.

Figure 2D-i shows a wide-field fluorescence image of a focused area on the ITO electrode. The full-time lapse movie of this area is displayed in Supplementary Video (Movie S1, Supplementary Information). Obviously, there were multiple rod-shape and micrometer-long microtubules per 80 × 80 µm fluorescence imaging area on the ITO electrode in the working buffer containing 3.6 nM cresyl violet and 0.2 mg/mL microtubule, where cresyl violet molecules were absorbed on microtubules and still some cresyl violet molecules were presented in solution and on the ITO electrode surface as well. One representative microtubule is selected and shown in zoomed-in images (Figure 2D-i and ii). Over the time period of 16 CV cycles (Figure 2A–C), fluorescence intensity of single molecules of cresyl violet stained on microtubules as well as those ones free in solution in the camera field of view were modulated by CV potential scanning (Supplementary Information/Supplementary Video, Movie S1), while cresyl violet molecules were turned to “Off” and “On” electrochemically. Examining single-molecule events of cresyl violet stained on microtubules from image to image over the 16 CV cycles, precise localizations of those single-molecule events were determined using STORM data processing method [26], [27], [28]. The blue dots are precise locations fitted for single molecules of cresyl violet stained on the selected microtubule with electrochemical modulation (Figure 2D-ii) (Supplementary Information/Supplementary Video, Movie S2). Correspondingly, the resulting high-resolution E-STORM images of the sample microtubule are shown in Figure 2D-iii. The resolution enhancement is further demonstrated in Figure 2D-iv, which displays one half imaged in conventional microscopy and another half imaged in E-STORM for the same microtubule. Figure 2E shows changes of total fluorescence intensity of cresyl violet stained on one of the sample microtubule (Figure 2D-ii and iii) over 16 continuous CV cycles, demonstrating that the fluorescence intensities of cresyl violet molecules on the microtubule were modulated synchronously with CV potential scanning as expected. After 16 CV cycles (Figure 2E), most of fluorescence intensity of cresyl violet on the microtubule remained, indicating that cresyl violet is a good candidate of redox fluorescent dye for E-STORM imaging with high photostability subject to electrochemical potential scanning.

It is noticed that there are a small fraction of the redox dye molecules stained on the protein filaments remained in the oxidized state even at the lowest potential as well, which could be resulted also from the anisotropic and unconstructive orientation. Since it has been confirmed that the reduced state of cresyl violet has no fluorescence [19], [20], although cresyl violet’s duty cycle is as high as 0.18, in this work, the “On” state of the majority of single molecules of cresyl violet on the protein filaments could be driven to “Off” state by electrochemical potential modulation and thus enable the SMLM imaging. The small fraction of those dye molecules remained in the oxidized state with unconstructive orientation at the negative potentials do not decrease the quality of the E-STORM imaging of the protein filaments because the probability to detect overlapped single molecules is low.

**Figure 3:**
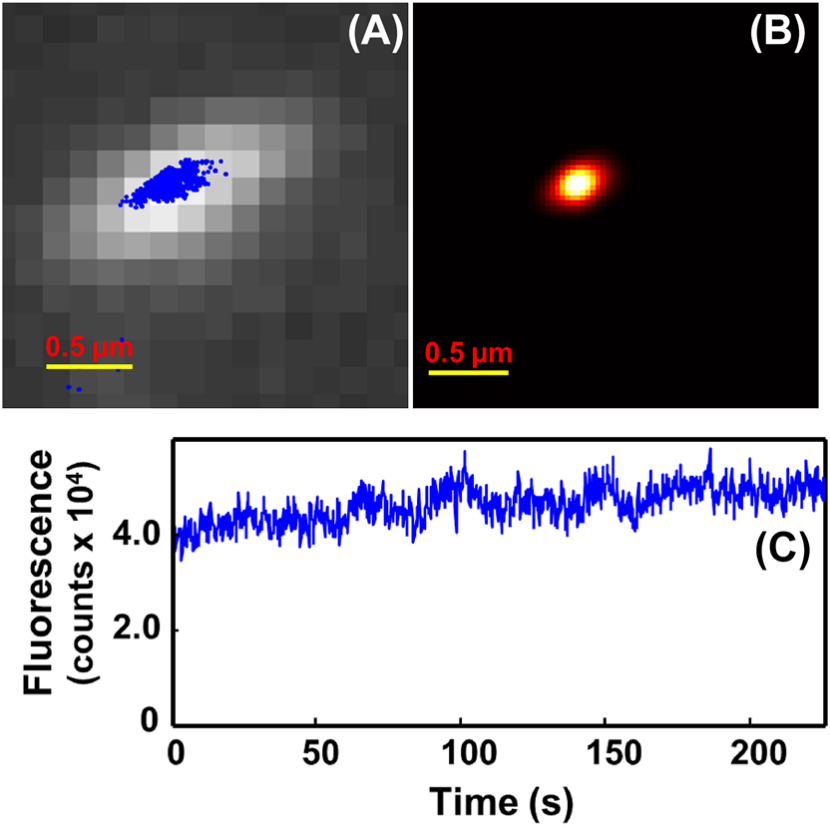
Fluorescence images and intensity-time trajectories of microtubule-cresyl violet. (A) Calculated single-molecule locations of microtubule-cresyl violet on the ITO electrode without electrochemical modulation are plotted as blue dots (Supplementary Video, Movie, Supplementary Information); (B) the image of microtubule-cresyl violet rendered from calculated single-molecule locations shown in (A); (C) plot of fluorescence intensity of microtubules-cresyl violet displayed in (A) versus time over the same time length as in (Figure 2E). The working buffer: 200 μL, pH 7.0, 15 mM PIPES containing 1 mM MgCl_2_ and 20 µM Taxol. [microtubule]: 0.2 mg/mL; [cresyl violet]: 3.6 nM.

Because of cresyl violet’s high duty cycle, without electrochemical modulation, there is high probability and likelihood that more than one cresyl violet molecule on the microtubule staying at “on” state at a time. To confirm electrochemical potential modulation contributes to single-molecule localization, control experiment was carried out over the same time length as running 16 continuous CV cycles (Figure 2A) but without electrochemical potential scanning. As a microtubule is as short as less than 1.0 μm–∼2 μm, the distance among multiple cresyl violet molecules on one microtubule could be very close, that says, within diffraction limit. However, the localization program has difficulty to distinguish multiple molecules with proximity within diffraction limit. The localization program thereby reported the center point of the multiple molecules as one location (Scheme 1), which caused major errors in the fitted localizations of cresyl violet on the control microtubule as exhibited in Figure 3A (Supplementary Information/Supplementary Video, Movie S3). Thus, the resulting image of the microtubule (∼0.4 μm) was much shorter than that of the true microtubule (∼2.0 μm) since the reported locations were mostly at the center region of the microtubules (Figure 3A and B). Figure 3C shows changes of total fluorescence intensity of cresyl violet stained on the control microtubule over the same time length as running 16 continuous CV cycles (Figure 2A). The fluorescence intensity remained at high level, indicating multiple cresyl violet molecules were at “ON state” on the microtubule.

SMLM requires well-resolved singe molecules in raw images. If single molecules are too close, the fitting program would consider them as one. It results in localization errors in SMLM. For processing SMLM data, avoiding spatially overlapping of single molecules is critical. For a microscopic object, the number of single molecules which can be recognized for position tracking by data processing software is limited. The acquired data should be well below this limit to reduce the error of single molecule localization. In a microscopic object, a microtubule in this work, there might be several molecules at “On” state within an image. If the number of molecules was low, the molecules were sparse in image object, then the chance of molecular overlapping was low. As more molecules were at “On” state, the software would identify more molecules. But eventually, if there were too many molecules at “On” state, the software would not be able to identify all of them due to overlapping issue. The reported number of molecules by software would reach a limit or a saturation. SMLM should not run in such a regime because the significant errors would occur in locating molecules. However, the saturation number is difficult to measure experimentally. The saturation number is related to the imaging object’s size and shape, image signal, background, noise levels, optical resolution, software algorithm and parameter settings, etc.

**Figure 4:**
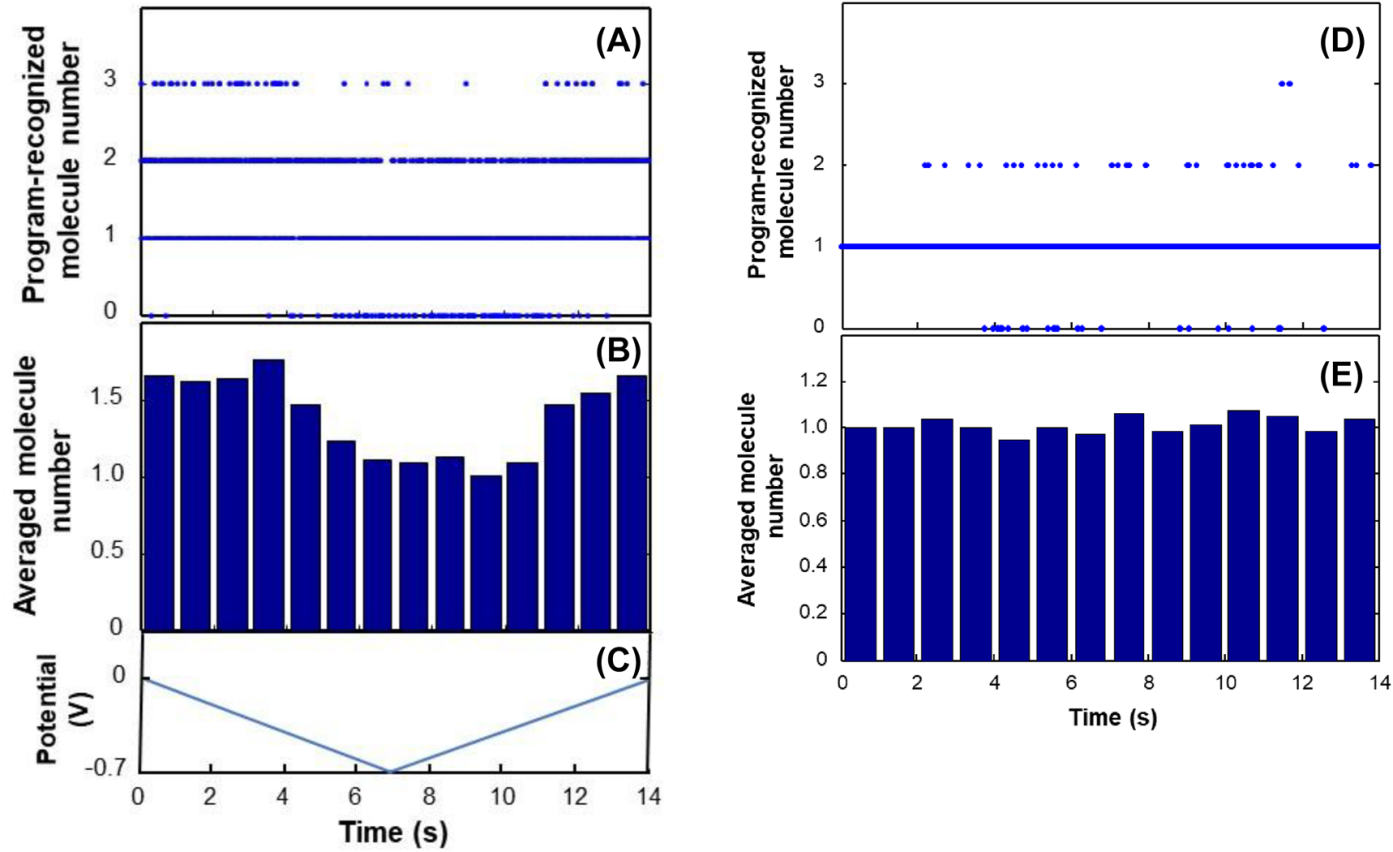
STORM program-recognized and averaged molecule number of cresyl violet per camera exposure with and without electrochemical potential scanning. (A) STORM program-recognized molecule number of cresyl violet per camera exposure on the sample microtubule (Figure 2D) while cyclic voltammetry was carried out by electrochemical potential scanning 16 cycles from 0.0 V to −0.7 V–0.0 V at a scan rate of 0.1 V/s for which all 16 cycles were grouped into one cycle’s time period from 0 s to 14 s; (B) averaged molecule number of cresyl violet detected per camera exposure on the microtubule for every 1-s time bin over the potential scanning (Figure 2A, C and D) while cyclic voltammetry was carried out by electrochemical potential scanning 16 cycles from 0.0 V to −0.7 V–0.0 V at a scan rate of 0.1 V/s for which all 16 cycles were grouped into one cycle from 0 s to 14 s; (C) plot of potential (*V*) versus time for one cycle of 16 continuous CV scans; (D) STORM program-recognized molecule number of cresyl violet per camera exposure on the control microtubule in the absence of electrochemical potential cycling. Data were collected and processed in the same time length and steps as done with the sample microtubule for (A) as if there were 16 cycles of 14 s; (E) averaged molecule number of cresyl violet per camera exposure for every 1-s time bin on the control microtubule in the absence of electrochemical potential cycling. Data were collected and processed in the same time length and steps as done with the sample microtubule for (A) as if there were 16 cycles of 14 s.

With the help of electrochemical potential modulation, in this work, we took a deeper insight into the molecule number saturation issue (Figure 4A–C). We have thus analyzed the images of the selected microtubule (Figure 2D-i and ii) under electrochemical modulation to determine if the number of molecules at “On” state is below the software limit. All camera exposures of the selected microtubule were examined (Figure 2D-i and ii). As camera exposure was synchronized with electrochemical modulation, we plotted the number of the recognized molecules on the selected microtubule for every acquired image (per camera exposure) versus the modulation time (correspondingly the potential) all within one cycle, while cyclic voltammetry was carried out by electrochemical potential scanning 16 cycles from 0.0 V to −0.7 V–0.0 V at a scan rate of 0.1 V/s for which one cycle took from 0 s to 14 s. Therefore, the horizontal axis of the plot was wrapped to 14-s time period corresponding to the electrochemical potential scanning of one cycle.

We can see that when the electrochemical potential was high (around 0.0 V), more molecules were recognized; when the electrochemical potential was low (around −0.7 V), fewer molecules were recognized (Figure 4A–C). From the selected microtubule, the software picked up 0, 1, 2, or 3 molecules per image. The plot shows that at higher potential time period, more molecules (∼2 or 3) were detected and at lower potential time period, fewer molecules (∼1 or 2) were detected. To clearly illustrate the correlation and change of the recognized molecule number with the electrochemical potential scanning, we chose 1-s time bin and averaged the data points per camera exposure in the 1-s time bins. Figure 4B shows the averaged molecule numbers that are recognized over the electrochemical potential scanning. At high electrochemical potential, there are about 1.6 molecules detected, while at low electrochemical potential, there is about 1 molecule detected. The change in the recognized molecule number on the microtubules over the electrochemical potential scanning is consistent with the switching of dark/bright states of cresyl violet by the redox reaction on the electrode (Supplementary Information/Supplementary Video, Movie S1). Higher potential would drive the molecules to the oxidized fluorescent state so more molecules would be in the “On” state. On the other hand, lower potential would drive the molecules to the reduced nonfluorescence state so fewer molecules would be observed. The number of detected molecules would provide the information about detection saturation. As the software could pick up as more and less molecules as the electrochemical potential was cycling from high to low, the data processing software was not saturated by the crowdedness of molecules.

The same analysis process was carried out for the control experiment as it was done with electrochemical modulation for single molecule imaging. We imaged a control microtubule without electrochemical modulation over the same time length as with electrochemical modulation. We also plotted the number of detected molecules versus time over the same time length based on one cycle time of 14 s (Figure 4D and E). Because no electrochemical modulation was applied, the number of detected molecules remained mostly constant. In contrast to that with electrochemical modulation, the localization software can only detect about “one” molecule per image on the microtubule in control experiment (Supplementary Information, Figure 4D and E). Since there was no electrochemical potential modulation, it is understandable that the average detected molecule number did not change with time (Figure 4D and E). More importantly, the software-detected “one” molecule per image does not mean there was only one cresyl violet molecule on the control microtubule at “On” state. The high fluorescence intensity and nonblinking behavior demonstrate there were many molecules on the control microtubule at “On” state because of the high duty cycle of cresyl violet. In the absence of electrochemical modulation, one observed fluorescence spot, which the software treated as “one” single molecule for location tracking, was actually from multiple molecules on the control microtubule (Figure 4D and E). From many indications, such as the rendered image (shortened) and nonblinking of the molecules on the control microtubule, we also believe the detected “one” molecule was not a single molecule, but multiple overlapped molecules. The reconstructed super-resolution image shows a shorter tube, not matching with the raw image at all (Figure 3A and B).

**Figure 5:**
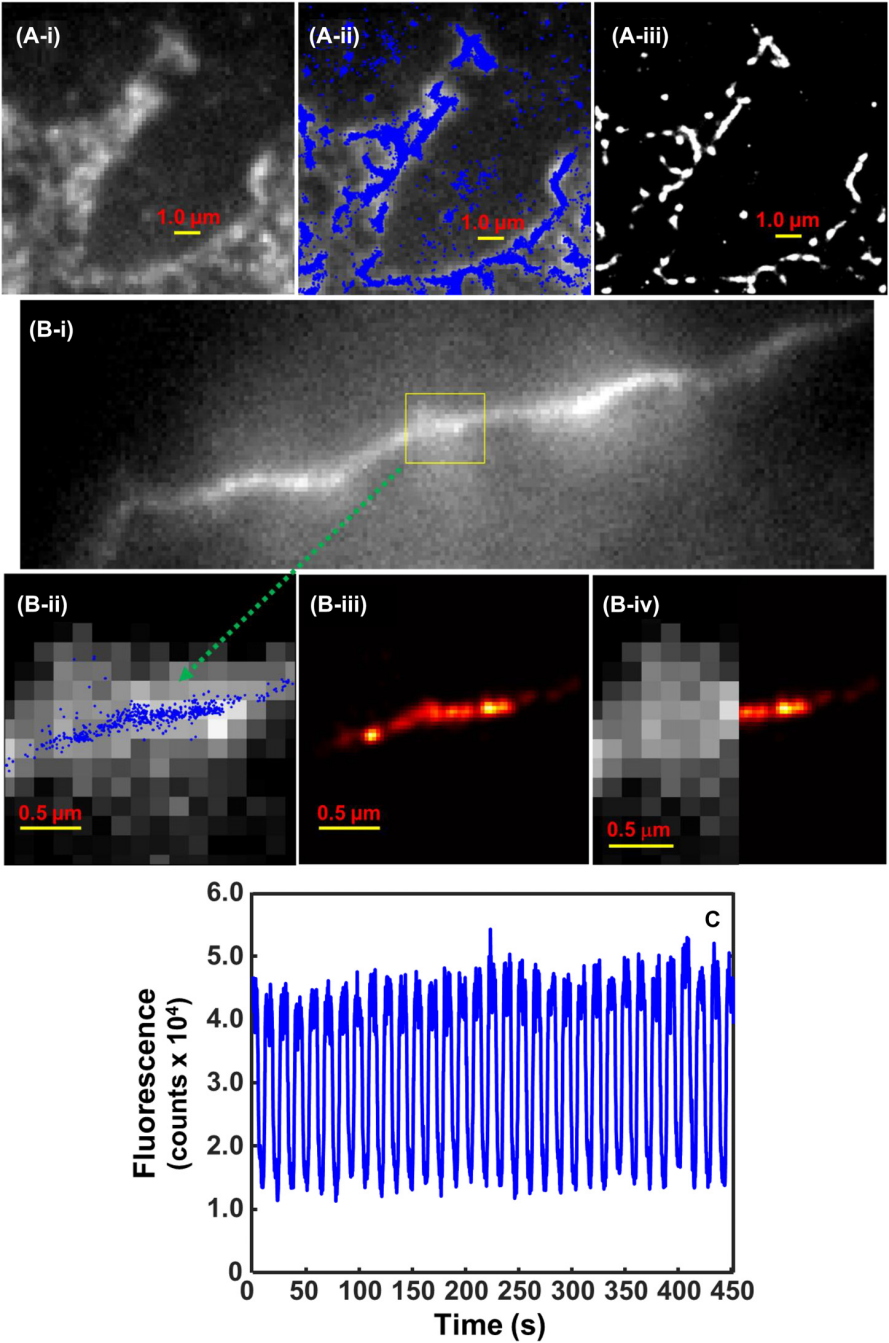
Fluorescence images and intensity-time trajectories of actin-cresyl violet with cyclic electrochemical potential scanning at the ITO electrode in the working buffer. (A) The wide-field fluorescence image of dense and crossed filaments of actin-cresyl violet on the ITO electrode with electrochemical modulation (Supplementary Video, Movie S4, Supplementary Information) (i); precise localizations of cresyl violet stained on the actin filaments plotted as blue dots (ii), the resulting super-resolution image of the actin filaments from rendered single-molecule positions (iii); (B) the wide-field fluorescence image of one long filament of actin-cresyl violet on the ITO electrode with electrochemical modulation (Supplementary Video, Movie, Supplementary Information) (i); precise localizations of cresyl violet stained on the selected part of the actin filament plotted as blue dots (ii), the resulting super-resolution image of the selected part of actin from rendered single-molecule positions (iii), and comparison of one half imaged in conventional microscopy and another half imaged in E-STORM to show the resolution improvement (iv); (C) plot of fluorescence intensity of actin-cresyl violet displayed in (A-ii) versus time during 32 continuous CV scans; the working solution: 165 μL of pH 6.2, 6 mM sodium phosphate and 35 μL of the diluted polymerization buffer (*Experimental Section*). [actin]: 0.063 mg/mL; [cresyl violet]: 8.64 nM; scan rate: 0.1 V/s; scan numbers: 32 cycles; potential scan range: 0.0 V to −0.7 V.

To further prove the concept of E-STORM, Figure 5A-i **shows** the conventional wide-field fluorescence image of dense and crossed filaments of actin-cresyl violet on the ITO electrode with electrochemical modulation (Supplementary Video, Movie S4, Supplementary Information). The filaments of actin-cresyl violet were prepared in the working solution containing 8.64 nM cresyl violet and 0.063 mg/mL actins (*Experimental Section*). There were multiple actin filaments with different polymerization degrees crossed or neighbored in the fluorescence imaging area on the ITO electrode (Figure 5A-i), where cresyl violet molecules were absorbed on the actin filaments and still some cresyl violet molecules were presented in solution and on the ITO electrode surface as well. The precise localizations of cresyl violet stained on the actin filaments are plotted as blue dots in Figure 5A-ii; thus, the super-resolution image of the actin filaments are resulted from rendered single-molecule positions (Figure 5A-iii). Figure 5B-i shows the wide-field fluorescence microscopy image of one long filament of actin-cresyl violet on ITO electrode. Its full-time lapse movie is shown in Supplementary Video (Movie S5, Supplementary Information). Over the time period of 32 CV cycles, fluorescence intensity of single molecules of cresyl violet stained on the short and lengthy actin structures in the camera field of view was modulated by CV potential scanning (Supplementary Information/Supplementary Video, Movies S4 and S5), while cresyl violet molecules were turned to “Off” and “On” electrochemically. Examining single-molecule events of cresyl violet stained on actins from image to image over the 32 CV cycles, precise localizations of those single-molecule events were determined using STORM and DAOSTORM data processing method [26], [27], [28], [36]. One selected part of the long actin structure is shown in zoomed-in images (Figure 5B-ii). The blue dots are precise locations fitted for single molecules of cresyl violet stained on the selected part of the actin structure with electrochemical modulation (Figure 5B-ii). Correspondingly, the resulting high-quality E-STORM image of the selected part of the actin structure is shown in Figure 5B-iii. The resolution enhancement is further demonstrated in Figure 5B-iv, which shows one half imaged in conventional microscopy and another half imaged in E-STORM for the same part of actin filament. Figure 5C shows changes of total fluorescence intensity of cresyl violet stained on the actin structure over 32 continuous CV cycles, demonstrating that the fluorescence intensities of cresyl violet molecules on the microtubule were modulated synchronously with CV potential scanning as expected. After 32 CV cycles (Figure 5C), the ensembled fluorescence intensity of cresyl violet on the actin structure was stable, confirming high photostability of cresyl violet stained on actins subject to electrochemical potential scanning. Cresyl violet has been studied for interfacial electron transfers by single molecule spectroscopy in earlier times [21], [37].

In addition, Figure S1A–F compared the images of actin-cresyl violet with and without electrochemical potential scanning (Supplementary Video, Movies S6 and S7, Supplementary Information). Figure S1D and E shows the image of the actin-cresyl violet without the electrochemical potential scanning was shortened and “fattened” compared to that with the electrochemical potential scanning (Figure S1A and B), although both the resolutions calculated from Fourier ring correlation were about 50–60 nm [38]. Without electrochemical modulation, the overlapped single molecules are counted as a spot under camera (Scheme 1). That spot can be localized also with good resolution under fitting algorithm, but it was not a true single molecule location. Consequently, the localization-based image from high duty cycle molecules could be distorted as discussed in the literature [16]. The result again confirmed that electrochemical potential scanning helped solve the molecule overlapping issues and reduce the localization errors and accordingly improve the imaging quality of the cytoskeletal protein structure as Scheme 1 demonstrated.

We have noted that there have been published localization software that can handle higher density single molecule images [36], [39]. These programs can alleviate the error of crowding to a degree but cannot solve the problems for unresolvable single molecules and, therefore, does not change the fundamental requirement that SMLM imaging needs fluorophores with low duty cycle or an efficient way to bring those nearby molecules from “On” state to “Off” state.

## 4 Conclusions

In conclusion, conventional super-resolution SMLM requires low duty cycle of fluorescent molecules to obtain well-separated single molecules on fluorescence images. However, many fluorescent dyes molecules do not have low enough duty cycles. We have demonstrated that, although a redox active fluorescent dye with high duty cycle is not suitable for conventional SMLM imaging, its “On/Off” state was able to be electrochemically modulated and thus the dark states of the fluorophore could be well controlled allowing the molecule overlapping issues and thus the localization errors to be minimized, accordingly electrochemical SMLM imaging became achievable. E-STORM requires fluorophores approaching to the electrode for effective electrode reaction [34], either in short distance or with the help of conductive medium. In this work, most of cresyl violet molecules on actin and microtubules were reduced and oxidized in response to the electrochemical potential scanning. Future cellular imaging by E-STORM may require conductive medium. E-STORM imaging of intracellular structure *in vivo* is ongoing in this laboratory. This method may pave the way for using several more classes of conventional redox-active fluorescent dyes including phenazines, phenoxazines, and phenothiazines and developing new redox fluorophores for SMLM imaging.

## Supplementary Information

Supplementary online materials and videos. The videos in AVI format were generated by ImageJ and can be viewed by Media Player.

## Supplementary Material

Supplementary Material Details

## References

[1] Betzig E. (2006). Imaging intracellular fluorescent proteins at nanometer resolution. *Science*.

[2] Andresen M. (2008). Photoswitchable fluorescent proteins enable monochromatic multilabel imaging and dual color fluorescence nanoscopy. *Nat. Biotechnol.*.

[3] Tiwari D. K., Nagai T. (2013). Smart fluorescent proteins: innovation for barrier-free superresolution imaging in living cells. *Dev., Growth Differ.*.

[4] Nienhaus K., Nienhaus G. U. (2014). Fluorescent proteins for live-cell imaging with super-resolution. *Chem. Soc. Rev.*.

[5] Zhuang X. (2016). Illuminating biology at the nanoscale with single-molecule and super-resolution imaging. *FASEB J.*.

[6] Bintu B. (2018). Super-resolution chromatin tracing reveals domains and cooperative interactions in single cells. *Science*.

[7] Rust M. J., Bates M., Zhuang X. (2006). Sub-diffraction-limit imaging by stochastic optical reconstruction microscopy (STORM). *Nat. Methods*.

[8] Bates M., Huang B., Dempsey G. T., Zhuang X. (2007). Multicolor super-resolution imaging with photo-switchable fluorescent probes. *Science*.

[9] Li H., Vaughan J. C. (2018). Switchable fluorophores for single-molecule localization microscopy. *Chem. Rev.*.

[10] Wang L., Frei M. S., Salim A., Johnsson K. (2019). Small-molecule fluorescent probes for live-cell super-resolution microscopy. *J. Am. Chem. Soc.*.

[11] Jradi F. M., Lavis L. D. (2019). Chemistry of photosensitive fluorophores for single-molecule localization microscopy. *ACS Chem. Biol.*.

[12] Cnossen J. (2020). Localization microscopy at doubled precision with patterned illumination. *Nat. Methods*.

[13] Ostersehlt L. M. (2022). DNA-paint minflux nanoscopy. *Nat. Methods*.

[14] Miller H., Zhou Z., Shepherd J., Wollman A. J. M., Leake M. C. (2018). Single-molecule techniques in biophysics: a review of the progress in methods and applications. *Rep. Prog. Phys.*.

[15] Patterson G., Davidson M., Manley S., Lippincott-Schwartz J. (2010). Superresolution imaging using single-molecule localization. *Annu. Rev. Phys. Chem.*.

[16] Dempsey G. T., Vaughan J. C., Chen K. H., Bates M., Zhuang X. (2011). Evaluation of fluorophores for optimal performance in localization-based super-resolution imaging. *Nat. Methods*.

[17] Vaughan J. C., Jia S., Zhuang X. (2012). Ultrabright photoactivatable fluorophores created by reductive caging. *Nat. Methods*.

[18] Lu H. P., Xie X. S. (1997). Single-molecule spectral fluctuations at room temperature. *Nature*.

[19] Lei C., Hu D., Ackerman E. J. (2008). Single-molecule fluorescence spectroelectrochemistry of cresyl violet. *Chem. Commun.*.

[20] Lei C., Hu D., Ackerman E. (2009). Clay nanoparticle-supported single-molecule fluorescence spectroelectrochemistry. *Nano Lett.*.

[21] Biju V., Micic M., Hu D. H., Lu H. P. (2004). Intermittent single-molecule interfacial electron transfer dynamics. *J. Am. Chem. Soc.*.

[22] Yip W. T., Hu D. H., Yu J., Vanden Bout D. A., Barbara P. F. (1998). Classifying the photophysical dynamics of single- and multiple-chromophoric molecules by single molecule spectroscopy. *J. Phys. Chem. A*.

[23] Yu J., Hu D. H., Barbara P. F. (2000). Unmasking electronic energy transfer of conjugated polymers by suppression of O-2 quenching. *Science*.

[24] Dempsey G. T., Bates M., Kowtoniuk W. E., Liu D. R., Tsien R. Y., Zhuang X. (2009). Photoswitching mechanism of cyanine dyes. *J. Am. Chem. Soc.*.

[25] Lei C., Hu D. (2021). High throughput mapping of single molecules’ redox potentials on electrode. *Anal. Chem.*.

[26] Cui Y. Fluctuation localization imaging-based fluorescence in situ hybridization (fliFISH) for accurate detection and counting of RNA copies in single cells. *Nucleic Acids Res.*.

[27] Thompson R. E., Larson D. R., Webb W. W. (2002). Precise nanometer localization analysis for individual fluorescent probes. *Biophys. J.*.

[28] Hu D., Azevedo N., Almeida C. (2021). *Fluorescence In-Situ Hybridization (FISH) for Microbial Cells: Methods and Concepts*.

[29] Magde D., Brannon J. H., Cremers T. L., Olmsted J. (1979). Absolute luminescence yield of cresyl violet. A standard for the red. *J. Phys. Chem.*.

[30] Biranowska J., Berdel B., Ludkiewicz B., Dziewiatkowski J., Jagalska-Majewska H., Moryś J. (2000). Developmental changes of MAP2 immunoreactivity in the hippocampus proper and dentate gyrus of the rat. *Folia Neuropathol.*.

[31] Alvarezbuylla A., Ling C. Y., Kirn J. R. (1990). CRESYL violet – a red fluorescent NISSL stain. *J. Neurosci. Methods*.

[32] George M., Meining A. (2003). Cresyl violet as a fluorophore in confocal laser scanning microscopy for future in-vivo histopathology. *Endoscopy*.

[33] Takahashi N., Tarumi W., Hamada N., Ishizuka B., Itoh M. T. (2017). Cresyl violet stains mast cells selectively: its application to counterstaining in immunohistochemistry. *Zool. Sci.*.

[34] Bard A. J., Faulkner L. R. (2001). *Electrochemical Methods: Fundamentals and Applications, Edn. The 2cd*.

[35] Elgrishi N., Rountree K. J., McCarthy B. D., Rountree E. S., Eisenhart T. T., Dempsey J. L. (2018). A practical beginner’s guide to cyclic voltammetry. *J. Chem. Educ.*.

[36] Holden S. J., Uphoff S., Kapanidis A. N. (2011). DAOSTORM: an algorithm for high-density super-resolution microscopy. *Nat. Methods*.

[37] Chen R., Wu R., Zhang G., Gao Y., Xiao L., Jia S. (2014). Electron transfer-based single molecule fluorescence as a probe for nano-environment dynamics. *Sensors*.

[38] Koho S., Tortarolo G., Castello M., Deguchi T., Diaspro A., Vicidomini G. (2019). Fourier ring correlation simplifies image restoration in fluorescence microscopy. *Nat. Commun.*.

[39] Mukamel E. A., Babcock H., Zhuang X. (2012). Statistical deconvolution for superresolution fluorescence microscopy. *Biophys. J.*.

