# Supplementary material for: Electrochemically modulated single-molecule localization microscopy for in vitro imaging cytoskeletal protein structures
Source: Nanophotonics. 2025 Feb 10;14(4):459–70. doi: 10.1515/nanoph-2024-0559 (PMC11834056; doi:10.1515/nanoph-2024-0559)
Supplement: Supplementary file 1 — Supplementary Material Details [file j_nanoph-2024-0559_suppl_001.pdf]

Supplementary Information for

Electrochemically modulated single-molecule localization microscopy for *in vitro* imaging cytoskeletal protein structures

Chenghong Lei[1] and Dehong Hu[2]

[1]Guilin University of Technology, College of Chemistry and Bioengineering, Guilin, Guangxi 541006, China

[2]Environmental Molecular Sciences Laboratory, Pacific Northwest National Laboratory, Richland, Washington 99352, USA

Supplemental videos and figure

Movie S1. The recorded video displaying cresyl violet stained on microtubules (Figures 2D-i) on the ITO electrode surface going dark and bright in synchronization with 16 continuous CV scans. The size of the imaging zone was 80 x 80 μm. The working buffer: pH 7.0, 15 mM PIPES containing 1 mM $MgCl_2$ and 20 μM Taxol. [microtubule]: 0.2 mg/mL; [cresyl violet]: 3.6 nM; Scan rate: 0.1 V/s; Scan numbers: 16 cycles, Potential scan range: 0.0 V to -0.7 V.

Movie S2. The zoom-in video of Movie S1 displaying cresyl violet stained on the sample microtubule (Figures 2D-ii and 2D-iii) on the ITO electrode surface going dark and bright in synchronization with 16 continuous CV scans. The working buffer: pH 7.0, 15 mM PIPES containing 1 mM $MgCl_2$ and 20 μM Taxol. [microtubule]: 0.2 mg/mL; [cresyl violet]: 3.6 nM; Scan rate: 0.1 V/s; Scan numbers: 16 cycles, Potential scan range: 0.0 V to -0.7 V.

Movie S3. The zoom-in video displaying cresyl violet stained on the control microtubule on the ITO electrode surface without electrochemical modulation (Figures 3A and 3B). The working buffer: pH 7.0, 15 mM PIPES containing 1 mM $MgCl_2$ and 20 μM Taxol. [microtubule]: 0.2 mg/mL; [cresyl violet]: 3.6 nM.

Movie S4. The recorded video displaying cresyl violet stained on the actin filaments (Figures 5A-i, ii, and iii) on the ITO electrode surface going dark and bright in synchronization with 32 continuous CV scans. The size of the imaging zone was 11.2 x 11.2 μm. The working solution: 165 μL of pH 6.2, 6 mM sodium phosphate and 35 μL of the diluted polymerization buffer (*Experimental Section*). [actin]: 0.063 mg/mL; [cresyl violet]: 8.64 nM; Scan rate: 0.1 V/s; Scan numbers: 32 cycles; Potential scan range: 0.0 V to -0.7 V.

Movie S5. The recorded video displaying cresyl violet stained on the actin filament (Figures 5B-i, ii, and iii) on the ITO electrode surface going dark and bright in synchronization with 32 continuous CV scans. The size of the imaging zone was 29.9 x 9.4 μm. The working solution: 165 μL of pH 6.2, 6 mM sodium phosphate and 35 μL of the diluted polymerization buffer (*Experimental Section*). [actin]: 0.063 mg/mL; [cresyl violet]: 8.64 nM; Scan rate: 0.1 V/s; Scan numbers: 32 cycles; Potential scan range: 0.0 V to -0.7 V.

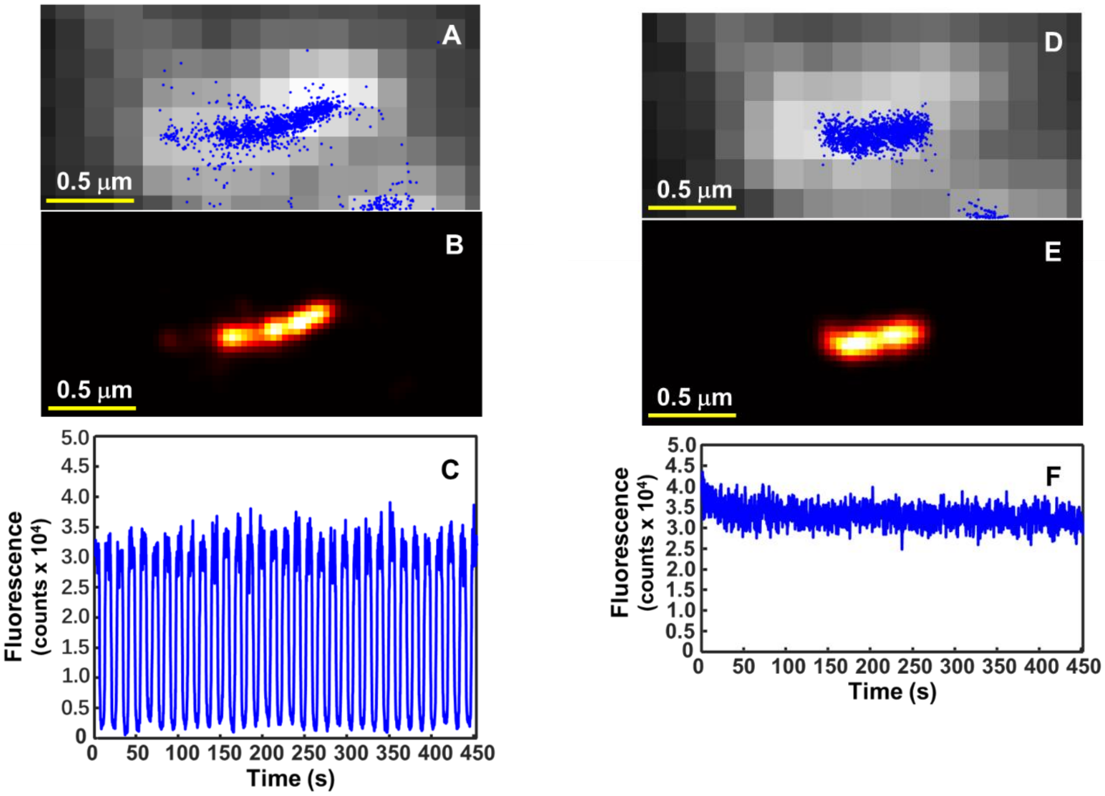

Figure S1. Fluorescence images and intensity-time trajectories of identical actin-cresyl violet with and without cyclic electrochemical potential scanning. (**A**) Calculated single-molecule locations of actin-cresyl violet on the ITO electrode with electrochemical modulation are plotted as blue dots (*Supplemental Video,* Movie S6, *Supplementary Information*); (**B**) The image of actin-cresyl violet rendered from calculated single-molecule locations shown in (**A**); (**C**) Plot of fluorescence intensity of actin-cresyl violet displayed in (**A)** vs. time during 32 continuous CV scans as in **Figure 5C**; (**D)** Calculated single-molecule locations of actin-cresyl violet on the ITO electrode without electrochemical modulation are plotted as blue dots (*Supplemental Video*, Movie S7, *Supplementary Information*); (**E**) The image of actin-cresyl violet rendered from calculated single-molecule locations shown in (**D**); (**F**) Plot of fluorescence intensity of actin-cresyl violet displayed in (**D**) vs. time over the same time length as in **Figure 5C**. The working solution: 165 μL of pH 6.2, 6 mM sodium phosphate and 35 μL of the diluted polymerization buffer (*Experimental Section*). [actin]: 0.063 mg/mL; [cresyl violet]: 8.64 nM; Scan rate: 0.1 V/s; Scan numbers: 32 cycles; Potential scan range: 0.0 V to -0.7 V.

Movie S6. The zoom-in video displaying cresyl violet stained on the actin filament on the ITO electrode surface going dark and bright in synchronization with 32 continuous CV scans (**Figures S1A, S1B and S1C**). The working solution: 165 μL of pH 6.2, 6 mM sodium phosphate and 35 μL of the diluted polymerization buffer (*Experimental Section*). [actin]: 0.063 mg/mL; [cresyl violet]: 8.64 nM; Scan rate: 0.1 V/s; Scan numbers: 32 cycles; Potential scan range: 0.0 V to -0.7 V.

Movie S7. The zoom-in video displaying cresyl violet stained on the same actin filament for **Figures S1A and S1B** on the ITO electrode surface without electrochemical modulation (**Figures S1D and S1E**). The working solution: 165 μL of pH 6.2, 6 mM sodium phosphate and 35 μL of the diluted polymerization buffer (*Experimental Section*). [actin]: 0.063 mg/mL; [cresyl violet]: 8.64 nM.
